# New Arthropod Containment Recommendations Provide Essential Guidance for Safety of Gene Drive Research

**DOI:** 10.4269/ajtmh.21-1148

**Published:** 2021-11-30

**Authors:** Stephanie James, David O’Brochta

**Affiliations:** Foundation for the National Institutes of Health, North Bethesda, Maryland

Recent research increasingly supports the potential for gene drive systems as innovative and cost-effective tools for controlling or eliminating important vector-borne diseases such as malaria and dengue, by introducing useful transgenes into target vector populations.^1^ Gene drive favors the inheritance of certain genes from generation to generation, resulting in those genes becoming increasingly prevalent in the population.^2^ Introduction of transgenes that decrease vectorial capacity, for example by inhibiting vector reproduction and/or limiting pathogen development within the vector, could have a major impact on pathogen transmission, thereby reducing human disease. Because gene drive technologies have not yet been field tested, however, there are no data on the possible environmental or health effects of releasing gene drive–modified organisms. For this reason, there have been widespread calls for additional guidance on risk assessment and management, and some have even proposed a moratorium on gene drive research until such guidance is in place.^3^

One immediate need has been for guidance on appropriate containment measures that researchers should follow when investigating gene drive–modified organisms, given that these are meant to spread their transgenes by interbreeding with compatible local species. For example, a 2020 survey of biosafety professionals^4^ revealed that the majority felt existing guidance was inadequate for making risk assessments and containment decisions regarding gene drive–modified arthropods. Lack of standard guidance can lead to uneven application of containment measures among institutions and decreased public confidence in the research.

The American Committee of Medical Entomology (ACME) of the American Society of Tropical Medicine and Hygiene has responded to this need with the recent publication of an addendum to its widely influential Arthropod Containment Guidelines.^5^ The new addendum^6^ provides specific recommendations on containment practices for arthropods modified with engineered transgenes capable of gene drive.

Several different gene drive systems have been proposed, for which perceived risk will vary. With some, spread is intended to be spatially or temporally limited, while with others, the modification is intended to spread widely through interbreeding populations and to persist indefinitely. Location of the research facility also will have a bearing, since breach of containment in areas where the environment is hospitable to survival of the escaped gene drive–modified arthropods would increase risk, particularly when the species already is present locally. The new ACME guidance^6^ takes a risk-based approach, recognizing that in some situations existing recommendations for containment of unmodified arthropods or those containing nondriving transgenes may be adequate. When risk assessment indicates the possibility of establishment of a new arthropod vector species or genetically modified arthropods in the local environment, the guidance recommends a new Arthropod Containment Level 2+ (ACL 2+) that expands on ACL 2, which has been endorsed for other genetically modified vectors. The guidance proposes questions to be addressed in risk assessment to help biosafety officials make this distinction. These questions examine, for example, the type of technology that results in gene drive, the location of the research, the expected results of the modification, and the possible public health or environmental hazards that might ensue from breach of containment.

Recommendations for ACL 2+ build on prior ACL guidelines, focusing on additional considerations such as restrictions to laboratory access, segregation of workspaces, strain verification, and other best practices. The guidelines also provide specific advice on facility design and security, arthropod handling, containment monitoring, training, and response in the case of escape of a modified arthropod.

Authoritative guidance, such as this new addendum^6^ to the ACME Arthropod Containment Guidelines,^5^ the updated Guidance Framework for Testing Genetically Modified Mosquitoes published recently by the World Health Organization,^7^ and the earlier Gene Drives on the Horizon report by the National Academies of Science, Engineering, and Medicine^8^ is absolutely essential to ensure that gene drive research can be conducted safely and responsibly both within the United States and in other regions. With these recommendations, ACME has made a valuable and far-reaching contribution toward safeguarding the potential for promising new gene drive strategies to be appropriately developed and tested for their ability to contribute to the control of vector-borne diseases.
